# Adaptable, illumination patterning light sheet microscopy

**DOI:** 10.1038/s41598-018-28036-2

**Published:** 2018-06-25

**Authors:** Rory M. Power, Jan Huisken

**Affiliations:** 1 Medical Engineering, Morgridge Institute for Research, 330 N Orchard Street, Madison, Wisconsin 53715 USA; 2 Department of Biomedical Engineering, University of Wisconsin-Madison, 1550 Engineering Drive, Madison, Wisconsin 53706 USA; 3 Max Planck Institute of Molecular Cell Biology and Genetics, Pfotenhauerstrasse 108, 01307 Dresden, Germany

## Abstract

Minimally-invasive optical imaging requires that light is delivered efficiently to limit the detrimental impact of photodamage on delicate biological systems. Light sheet microscopy represents the exemplar in tissue specific optical imaging of small and mesoscopic samples alike. However, further gains towards gentler imaging require a more selective imaging strategy to limit exposure to multiple yet discrete tissues without overexposing the sample, particularly where the information content is sparse or particularly optically sensitive tissues are present. The development of sample-adaptive imaging techniques is crucial in pursuit of the next generation of smart, autonomous microscopes. Herein, we report a microscope capable of performing 4D (*x*, *y*, *z*, *t*) light patterning to selectively illuminate multiple, rapidly reconfigurable regions of interest while maintaining the rapid imaging speed and high contrast associated with light sheet microscopy. We illustrate this utility in living zebrafish larvae and phantom samples.

## Introduction

Biological imaging inherently requires one to make compromises regarding spatial and temporal resolution, achievable signal and the health of the sample. In pursuit of unambiguous biological findings, this last factor is particularly important, dictating that every photon should be used efficiently and ideally in a targeted manner^1^.

Light sheet fluorescence microscopy (LSFM) differs from more conventional fluorescence techniques in that it uses paired orthogonal optical pathways to confine illumination to a single plane, thus delivering intrinsic optical sectioning without over-exposing the sample. This approach has redefined long-term fluorescence imaging and allows routine imaging of small embryos over several days with minimal detriment^2^. The development of new mounting methods, allows more physiologically relevant imaging and for samples to traverse several developmental stages unhindered^3^. However, the photon load will ultimately provide a hard limit to specimen viability and even the gentlest imaging will begin to take its toll.

Since the selective plane illumination microscope (SPIM) reinvigorated light sheet microscopy in the modern era^4^, further progress towards gentler microscopy has been slow, perhaps due to the favorable comparison between SPIM and conventional techniques. However, exploring biological development unhindered requires that our microscopes are able to do more with less. Continued progress in this regard, requires the development of new kinds of smart microscopy, not restricted to self-correction^5^ but which encompass a plethora of autonomous functions to adapt imaging to a given sample and crucially minimize our impact on the system under study^1^.

There is a need to re-visit the way light is delivered to the sample such that the spatiotemporal light intensity can be adapted to a given sample and its processes. Notably, in heterogeneous populations (e.g. mutant/drug screens) a one size fits all imaging strategy may miss crucial detail or overexpose samples imaged *in toto*. Herein, we report a light sheet microscope capable of delivering targeted illumination to specific regions of interest (ROIs) without sacrificing the speed and contrast that makes light sheet techniques so powerful. The zebrafish, often studied using LSFM^6,7^ provides a powerful test system for such a microscope.

## Results

### Spatiotemporal control of illumination in light sheet microscopy

**Figure 1:**
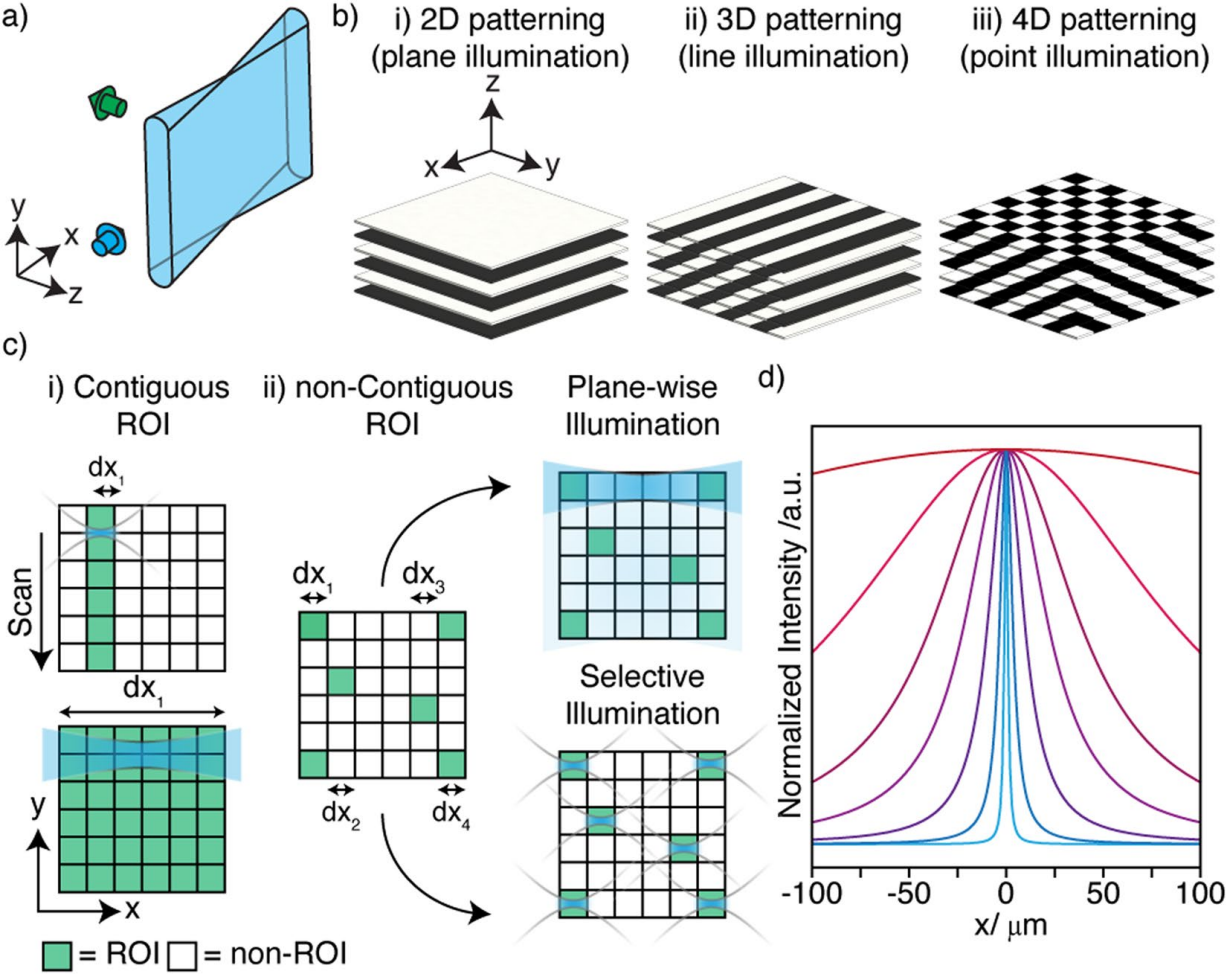
Selectively patterned illumination for light sheet microscopy. (**a**) The coordinate system used in relation to the light sheet throughout. Illumination and detection directions shown with blue/green arrows respectively (**b**) Degree of patterning achievable for different illumination modes. (**c**) Confining illumination to ROIs within the field of view (sub-divided by grid): (i) Conventionally, the length of the ROI (single region, d*x*_1_) comes to determine the light sheet length. (ii) For dynamic or non-contiguous ROIs (multiple regions, d*x*_n_) one may deliver illumination conventionally with some inherent overexposure or selectively to the individual ROIs. (**d**) On-axis profile of a Gaussian beam of varying NA_ill_ along the propagation axis (widest to narrowest distribution: NA = 0.02, 0.04, 0.06, 0.08, 0.12, 0.2, 0.4). Higher NA_ill_ leads to improved confinement of light and the most equitable spread of the incoming/outgoing light to the remainder of the tissue.

Consider the illumination scheme typical to SPIM. By convention, we denote the propagation direction, *x*, the light sheet height dimension, *y*, and the detection axis as *z* (Fig. 1a). 4D volumes (*x*, *y*, *z*, *t*) are captured as a time-sequence (*t*) comprising stacks (*z*) of 2D (*x*, *y*) optical slices. Since expression patterns during embryonic development may be spatially and temporally variant, this blind acquisition scheme resembles a brute force approach. To limit the deleterious effects of imaging, light should be delivered selectively only where and when necessary.

Naturally, some adaptation is possible between time-points, for example, to adapt exposure to temporally-variant expression patterns or indeed the temporal sampling itself. Likewise, within a single stack it is trivial to meter the light-dose on a plane by plane basis (Fig. 1b,i) to deliver 2D patterning (*z*, *t*). However, within the plane itself (*x*, *y*), the parallelized nature of the illumination, while a contributor to the low photodamage potential, leaves no room for adapting the distribution of light. This is unfortunate since sparsity is a feature of many biological systems where the information content of each plane may be far smaller than the whole. Indeed, many samples may contain tissues or structures that are particularly light sensitive and which should be avoided^8^. In this regard, illuminating with a static light sheet is inflexible and even wasteful.

A scanned (virtual) light sheet^9^ offers some respite, allowing patterning along the scan axis, *y* (Fig. 1b,ii) for 3D patterning (*y*, *z*, *t*) and has been used to produce reconfigurable periodic patterns used in structured illumination analogues^10^. We refer to this mode of light sheet production as 1D scanned throughout. Patterning along the light sheet propagation axis, *x*, which would allow full 4D patterning (*x*, *y*, *z*, *t*), however, remains a challenge (Fig. 1b,iii). To illuminate some sub-region, along the *x*-axis, d*x*, other on-axis structures that may not be similarly interesting are also illuminated (Fig. 1c). Conventionally, d*x* determines the desired field of view (FOV) and the light sheet length (determined by illumination numerical aperture, NA_ill_) (Fig. 1 c,i). However, when multiple but disperse sub-structures are of interest (several regions, d*x*_n_) or the size and position of d*x* changes with *y* position this scheme is inefficient since non-ROI areas are illuminated with an intensity approximately equal to that of that within the ROI (Fig. 1c,ii).

Although purely on/off patterning along *x* is not possible, a Gaussian beam spans many orders of magnitude in local intensity (intense focus but diffuse beam tails, Fig. 1d). This allows some degree of control over where light is delivered. Efficient patterning along *x* may therefore be considered to arise from two criteria. Firstly, matching the beam Rayleigh range, over which it may be considered approximately non-divergent, to the sub-ROI (d*x*_*n*_) length. In this way, the local intensity and spatial resolution throughout the region is effectively uniform. Secondly, the most equitable distribution of light through the sample on the way to and from the focus to ensure that regions excluded from the ROI experience the lowest possible peak intensities. These two criteria are a natural result of tuning NA_ill_ to match the size of the typical feature length (sub-ROI length) of the sample.

To understand how such an equitable distribution of light (throughout non-ROI areas) may be beneficial, consider the nature of photodamage. The rate of linear photodamage (arising from processes that scale linearly with the local intensity) is dependent only on the total light dose, which is independent of NA_ill_. However, photodamage is more commonly attributed to non-linear processes^11–15^ and hence scales non-linearly with the local intensity. Consider that beam divergence (in 1D) is proportional to NA_ill_^2^. For any point along the optical axis, the relative intensity (relative to the on-axis focus) will therefore scale as NA_ill_^−2^. Now assuming that the dominant order of optical process responsible for photodamage is second, the photodamage at this point will scale as NA_ill_^−4^ (i.e. the local intensity squared). Even when one factors in that the volume illuminated increases as NA_ill_^2^ the associated decrease in total non-linear photodamage would be expected to scale as NA_ill_^−2^. Correspondingly, a theoretical decrease in volume integrated photodamage should be possible by localization of the regions of high intensity to the ROI.

When one considers multiple sub-ROIs making up the full ROI, the increased localization of light comes at the expense of parallelization since all sub-ROIs cannot easily be illuminated simultaneously with the necessary selectivity (a discussion of strategies to effectively pattern along *x* is provided in Supplementary Note 1). In any case, simultaneous illumination of each sub-ROI would provide a mutually negative contribution to the overall image owing to the diffuse beam tails. For this reason, we eschew partially parallelized methods in favor of sequential illumination of sub-ROIs.

To cover the FOV, a short Rayleigh range beam (high NA_ill_ with respect to conventional light sheet microscopes) would have to be swept back and forth along *x* as it is simultaneously scanned in *y*. Relative to the 1D scanned light sheet, the drawback is that to maintain the signal-to-noise-ratio (SNR), the laser power must increase. The rate of useful signal production arises from the interplay between the region of the sample effectively probed and the probing intensity. We need only consider the cross-sectional area (*xy*) of the focal volume lying within the detection plane (*z* = 0). Since only this component of the focal volume elicits high contrast, in-focus signal, it is the scaling of this cross-section with NA_ill_ that is pertinent. Correspondingly, the effectively probed area scales as NA_ill_^−3^. The intensity scales as NA_ill_^2^ and so the overall scaling of the useful, in-plane signal rate is NA_ill_^−1^. How the two competing effects of: i) reduced energy density in non-ROI regions and ii) increased intensity required due to SNR constraints interact will depend to some degree on how much of the plane contains features to be imaged.

### Producing spatiotemporally patterned light sheets

A short line segment produced by focusing with moderate NA_ill_ does not constitute a light sheet unto itself and rather a virtual light sheet must be produced by sweeping the beam along *x* and scanning along *y*. By temporal control of the laser power, the resulting light sheet can, in principle, be patterned with a resolution governed by the beam parameters. Such an approach owes as much to random access point-scanning microscopies^16^ as to light sheet methods, although the focal volumes produced are much larger in this study (ca. 1000 μm^3^ compared to ca. 10 μm^3^ for typical point scanned microscopes, equating to a 100 times increased volumetric parallelization).

One of the major advantages of light sheet microscopy is its rapidity and it is crucial not to unduly sacrifice temporal resolution. 1D scanned light sheet variants are common and galvanometric mirrors are fast enough to rapidly scan the beam along *y*. However, the refocusing mechanism along *x* should be sufficiently fast as to cover the entire FOV and the light sheet constructed temporally within an exposure time of 10–25 ms. Mechanical refocusing with objective lenses is too slow to be viable, however, electrically tunable lenses have been used in light sheet microscopy to reposition a beam focus^17,18^. Unfortunately, such lenses are limited to operation at some tens of Hz^19^ and therefore do not allow in-plane patterning at anything approaching 10 s–100 s frames per second. Ferroelectric spatial light modulators have been used to refocus in less than 1 ms^20^, however, this still does not allow for a great deal of spatial patterning along *x* within the allotted exposure time. Instead, we employ a tunable acoustic gradient (TAG) index lens operating resonantly at 188 ± 1 kHz to allow rapid refocusing. This configuration has previously been shown to produce thinner light sheets with both linear^21,22^ and non-linear^23^ excitation and to allow remote focusing in the detection path^24^. Here, we employ a TAG lens and exploit the temporal dependence of the focal position and precise control of laser pulses to allow fully 3D patterned volumes, without sacrificing the rapid volumetric acquisition associated with LSFM. A full discussion of the temporal interplay between the refocusing system and laser modulation is given in Supplementary Note 2. We compare this imaging mode (referred to as 2D swept/scanned) to the 1D scanned mode.

In the absence of temporal constraints, the degree to which the sample volume can be patterned is governed only by the beam parameters and the interaction thereof with the inhomogeneous refractive index profile presented by the sample. We employ the rolling shutter and line-wise readout of an sCMOS camera as a spatial filter synchronized to the beam scanning (*y*)^25^ to remove diffuse signal from the swept beam tails such that they do not compromise contrast in the resulting image. Operating the camera in this manner has the dual role of removing the majority of the undesired background as well as non-ballistic signal arriving on the masked area of the camera.

### Patterning in optically homogeneous/inhomogeneous media

**Figure 2:**
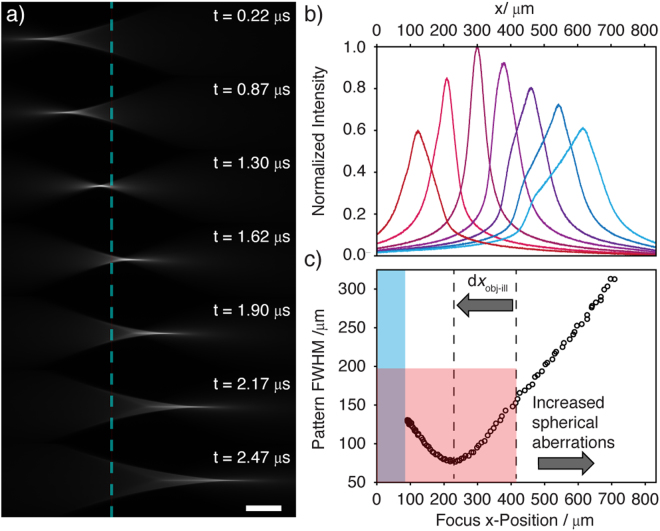
Characterization of the refocusing and propagation axis (*x*) patterning ability of the 2D swept/scanned mode. (**a**) Images of the beam (viewed in fluorescence) at various phases in the TAG lens cycle (61% TAG amplitude). The beam intensity is the most well-confined along *x* nearest to the native focus (i.e. with the TAG lens off), the position of which is shown by the blue dotted line. Scale bar: 100 μm. (**b** The beams shown in a) were scanned (y) and imaged with a confocal slit. The *y*-averaged on-axis profile of the illuminating light sheet is displayed, which defines the apparent patterning resolution in *x*. (**c**) The apparent pattern resolution as given by the intensity full-width at half maximum (FWHM) for different focal positions across the FOV (measured by the intensity maximum). Regions corresponding to the useful patterning range (red) and where the FWHM limit lies outside of the FOV (blue) are shown. The illumination objective was retracted by the distance shown from the center of the FOV.

Figure 2a shows the beam profile captured by pulsing the laser on (for 25 ns) at specific phases of the TAG lens. These images allow the spatially dependent beam profile to be viewed as a function of the TAG lens optical power (and hence refocus position). It was generally observed that the beam maintains a sharp focus either side of the native focus (i.e. with the TAG lens turned off). However, there was significant asymmetry about this position. To explore how the beam shape changed, the illumination objective was positioned such that its working distance lay approximately two-thirds of the way across the FOV (rather than being centered). During the 5.3 μs resonant period, the beam crosses the native focus twice. The beam profile shown for t = 1.3 μs, corresponds approximately to the native focal position. Away from this position, spherical aberrations increasingly lengthen the focus. This effect has been exploited to produce an extended depth of field (DOF)^26^ but here the influence is detrimental, reducing the ability to pattern effectively. Figure 2b shows the on-axis beam profile for different focal positions across the FOV. Measuring the *x*-axis patterning resolution from the images is difficult; each 2D image will actually be a *z*-projection of the 3D beam profile (out of focus regions contribute equally to signal albeit more diffusely). Consequently, the intensity as viewed from the image will not fall as rapidly away from the focus along *x* as would otherwise be the case. However, light penetrates only a few hundred μm into all but the most transparent tissues and the patterning ability far from the native focal position is rarely important.

Additionally, the modulation bandwidth places a small but non-negligible constraint on the patterning resolution (Supplementary Note 2.) Figure 2c shows the full width at half maximum (FWHM) apparent *x*-patterning resolution from the images. The desired patterning resolution is highly sample dependent but for the mm-sized zebrafish embryo 200 μm is reasonable to cover entire tissues. Using this as a definition of a useable patterning resolution still allows a FOV > 500 μm. The measured resolution may be considered as a worst-case scenario and it will shortly be shown that the actual *x*-axis patterning resolution is likely to be far superior.

**Figure 3:**
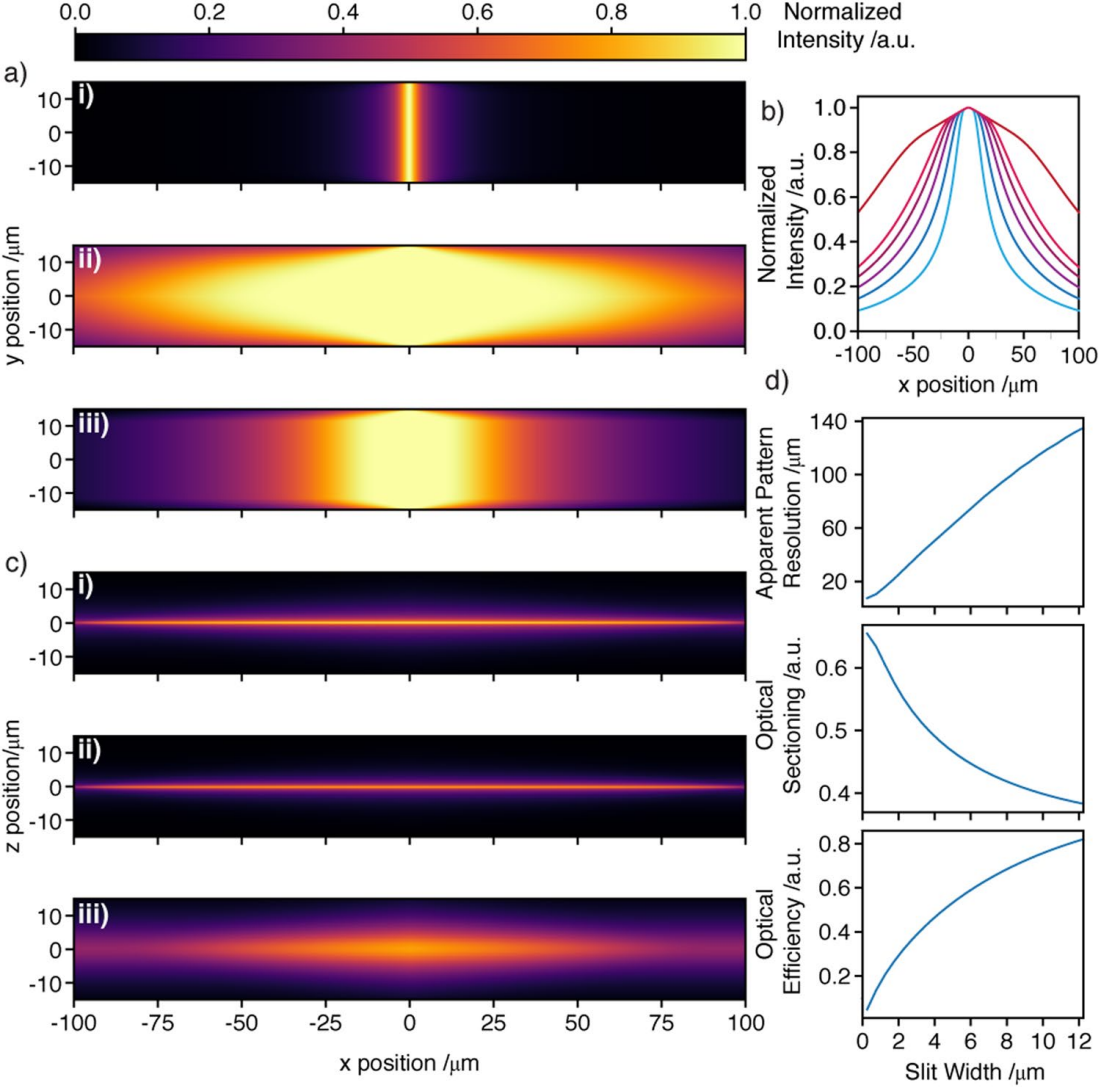
Light sheet simulations (NA_ill_ = 0.3, immersion media refractive index, n_imm_ = 1.33, λ_ill,0_ = 488 nm). (**a**) The *x*, *y* intensity profile of (i) a *y*-scanned light sheet (*z* = 0 μm). (ii) The *z*-projection of the light sheet. (iii) The z-projection of the light sheet using a confocal slit 7.25 μm wide (confocal slit widths are defined in object space throughout). (**b**) *y*-averaged intensity along the propagation axis of the *z*-projected light sheet (apparent patterning resolution in (*x*) from (**a**) (iii) with a varying slit width (from widest to narrowest: widefield (no-slit), 3.25, 5.25, 7.25, 9.25 and 11.25 μm). (**c**) The *x*, *z* profile of (i) the *y*-projection of a *y*-scanned light sheet. (ii) The same but using a confocal slit 7.25 μm wide. (iii) The light rejected by the confocal slit (6 × intensity shown for ease of visualization). (**d**) Apparent patterning resolution (FWHM) from the *z*-projected light sheet, optical sectioning and optical efficiency for various slit widths.

As stated, the actual patterning resolution along *x* is more difficult to determine from images. Instead, a set of simulations illustrate some points regarding apparent patterning and explore how the confocal slit width (as produced by the rolling shutter of the camera) comes to affect image quality and optical efficiency. Details of the simulations are provided in Supplementary Note 3. Figure 3(a) is illustrative of the former point: i) shows the *x*, *y* slice through the *z* center of the resulting intensity profile and illustrates the in-plane *x*-patterning resolution one would expect to achieve. Clearly, this is superior (FWHM = 7.5 ± 0.5 μm) than that observed in Fig. 2 (FWHM = 76.8 ± 0.8 μm at the native focus). Figure 3a,ii shows the z-projected profile in the absence of the confocal slit and iii provides a more realistic estimate of the pattern resolution observed from images at the native focus (FWHM = 89 ± 0.5 μm) and illustrates that the in-plane pattern resolution using the 2D swept/scanned mode is higher than observed in Fig. 2. For this reason, we refer to the apparent patterning resolution throughout when considering the pattern resolution measured from images.

The ability to deliver illumination to specific ROIs while maintaining good image quality is dependent on efficient removal of the out of plane, diffuse contribution to signal from the beam tails as they too are swept across the FOV. Failure to do so would limit the apparent ability to perform the patterning (from images) and result in low contrast images. To some extent each will be determined by the width of the confocal slit. Figure 3b shows the y-averaged intensity profile along *x* as a function of slit width (i.e. the apparent *x* patterning resolution).

While the confocal slit parameters do not influence the patterning resolution within the sample they do influence the perceived measure thereof. The loss of contrast provides a more challenging issue to address in any case. To explore the effect of sweeping the beam (*x*) a second set of simulations was carried out. Figure 3c shows the *y*-projected profile of the beam for (i) in the absence of the confocal slit, (ii) with the confocal slit and (iii) the light rejected by the confocal slit. While the slit clearly removes some of the signal arising from the focal plane (*z* = 0) it also efficiently removes the out of focus component.

A narrower slit will provide superior sectioning but reduces optical efficiency (more photons rejected). We now explore the apparent patterning resolution, optical sectioning and optical efficiency as a function of the slit width. The former has been defined already, while the optical sectioning is calculated as the fraction of the integrated intensity lying within the range: -DOF_det_ < *z* < DOF_det_, where DOF_det_ is the detection depth of field (i.e. objects remain in focus within ± DOF_det_) and which is calculated assuming NA_det_ = 0.8, λ_det,0_ = 510 nm, n_imm_ = 1.33:

1 $$DO{F}_{\det }=\frac{{{\lambda }}_{\det ,0}.{n}_{imm}}{N{{A}_{\det }}^{2}}.$$

Optical efficiency is simply given by the fraction of light passed by the confocal slit across the FOV. Figure 3d illustrates that (i) apparent pattern resolution and (ii) optical sectioning worsen as the slit is made wider.

Conversely the optical efficiency increases. Previous studies have shown that the 2D swept/scanned scheme is capable of delivering exemplary axial resolution and sectioning^21^. However, this required the use of a very narrow slit (160–480 nm), with severe detriment to optical efficiency. Since our aim is rather to limit sample exposure, optical efficiency is paramount. In this regard, we adopt a slit width of 7.25 µm throughout to achieve optical efficiency of ca. 70%. We note that this is ca. 2 times wider than the static beam (1/e^2^ beam diameter). Empirically, this accounts for some broadening of the beam observed due to aberrations induced by the TAG lens as well as improved light throughput. Halving the slit width would provide only a 16% improvement in sectioning accompanied by a 33% decrease in optical efficiency. For comparison, a conventional 1D scanned light sheet with an equivalent length (NA_ill_ = 0.0454) delivers sectioning of 0.53, superior yet comparable to the 0.43 of the 2D swept scanned mode (using slit width = 7.25 μm). Moreover, in refractive/scattering media a narrow-slit limits depth penetration (where the slit and illuminating beam are no longer co-aligned).

**Figure 4:**
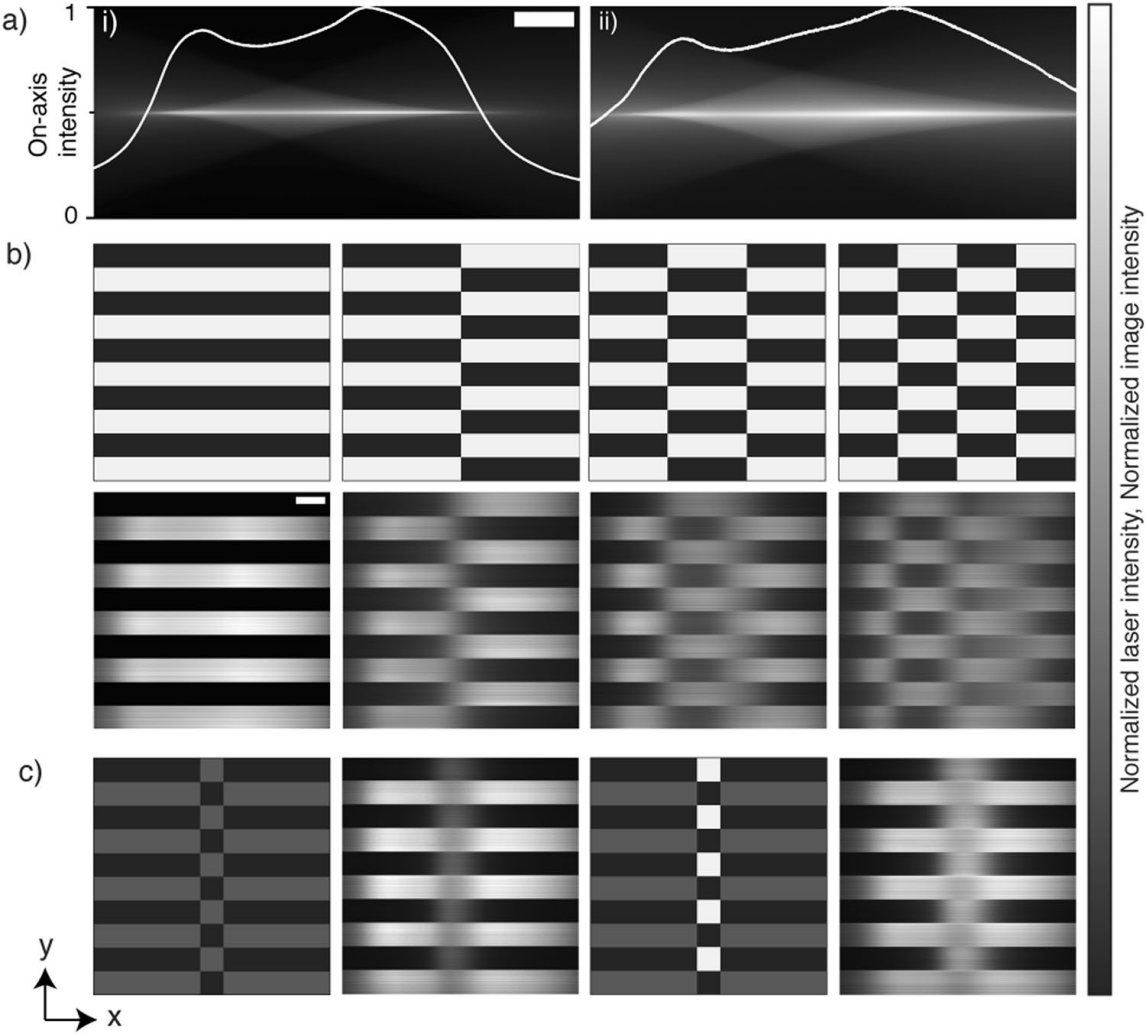
Illumination patterning for the 2D swept/scanned mode. (**a**) The fluorescent signal evolved by sweeping a focused Gaussian beam through a dye solution. (i) 42% TAG lens amplitude (ii) 61% TAG lens amplitude (98.4% of FOV within half-maximum intensity. The white line profiles show the swept beam intensity along the propagation axis. (**b**) Binary (digital) illumination patterns. Top: intended illumination pattern, bottom: resulting pattern seen in fluorescence (61% TAG amplitude). (**c**) Analog illumination patterns. First and third images: intended illumination patterns, second and fourth images: resulting pattern seen in fluorescence featuring duty cycle intensity correction (61% TAG amplitude). All scale bars: 100 μm.

Although the simulations have been carried out over a smaller *x* range than typically used (measured 2D swept/scanned total defocus range = 490 ± 0.8 μm, FWHM (*x*) = 572 ± 0.8 μm at 42% amplitude or 737 ± 0.8 μm (FWHM undefined) at 61% amplitude (Fig. 4a) owing to ease of computation, the behavior is generally applicable (optical sectioning will worsen in both the 1D and 2D cases). Having determined the appropriate slit parameters, Fig. 4b illustrates the apparent ability to produce desired illumination patterns. The failure to fully recapitulate the features of the patterns along *x* is a natural consequence of the non-zero length of the beam focus as well as the projected nature of the images, which blur the ideally discrete boundary between the on and off states. Nevertheless, the apparent patterning ability is good for low spatial frequency patterns (along *x*), gradually worsening as the number of cycles within the FOV increases. Clearly, the ability to pattern in *y* remains superior as would be expected.

Changes in the line-wise spatial duty cycle (in *x*) are naturally accompanied by changes in the fluorescence intensity owing to mutual contribution to signal from overlap of neighboring ‘points’ focal volumes. Having different regions experiencing different intensities is undesirable, at least without *a priori* knowledge of the fluorophore distribution. For example, small and sparsely labelled structures may go undetected, while large and densely labelled regions may saturate. The available analog modulation bandwidth provides the potential to perform line-wise (along *y*) intensity control either to correct these differences or to account for the fluorophore distribution, analogous to controlled light exposure microscopy^27^. For example, lines containing a large fluorophore concentration may be illuminated more dimly to account for the increased signal level, thus avoiding unnecessary exposure and saturation. Figure 4c illustrates this concept. The line-wise duty cycle was alternated between 10 and 90%. In the absence of analog modulation of the laser power the 10% duty cycle lines clearly appear much dimmer. Simply allowing the microscope to autonomously meter the illumination intensity according to the duty cycle allowed the intensities to be matched within ±20%. This residual mismatch is simply due to the sinusoidal motion of the focus (and resulting spatial dependence of dwell time, exemplified by the intensity profiles of Fig. 4a. In future, the sinusoidal dependence could be corrected using polarization optics^23^. Line profiles (along *x*) for each of the images are included in Supplementary Figure 1. It is trivial to extend this control of illumination intensity to *z* by selecting specific planes to image^28^ and temporally (*t*) by varying the time-lapse period to adjust for well temporally localized processes^29,30^.

**Figure 5:**
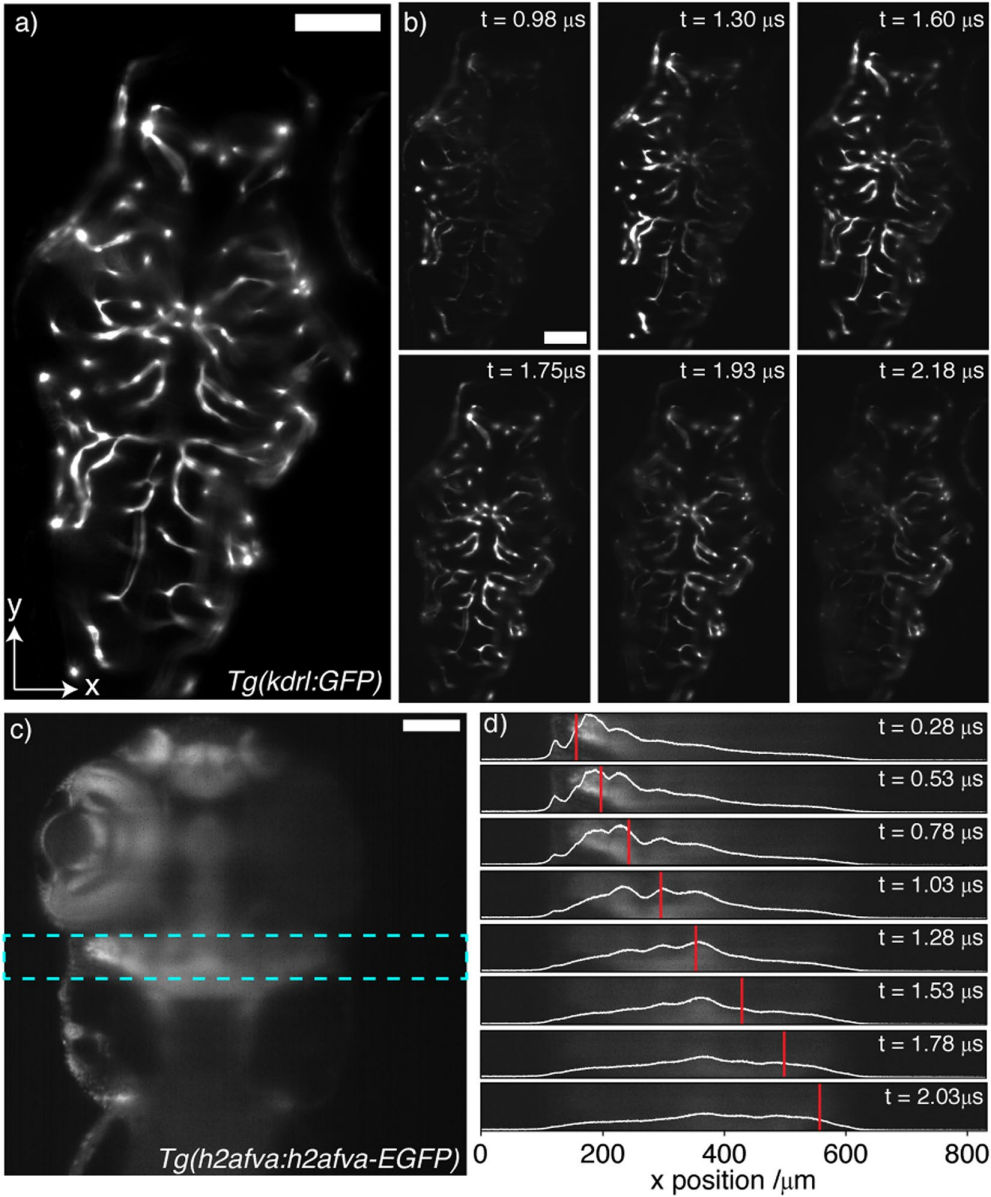
Illumination patterning in live zebrafish larvae (42% TAG amplitude). Scale bars = 100 μm. (**a**,**b**) *Tg*(*kdrl:GFP*) zebrafish (5 dpf), (**c**,**d**): Tg(h2afva:h2afva-EGFP) (5 dpf). (**a**) Fully illuminated plane through head and trunk section. (**b**) Patterning along x by phase-delayed illumination pulsing. (**c**) Fully illuminated plane through head and trunk section. Cyan box corresponds to ROI for (**d**). (**d**) Patterning along *x* by phase-delayed illumination pulsing. White line shows y-averaged illumination intensity. Red line shows position of beam focus for calibration performed in fluorescent dye solution.

Having demonstrated that the 2D swept/scanned approach is capable of illumination patterning in optically homogeneous media the same mode was characterized in a tissue context. The zebrafish embryo is frequently cited as being amenable to optical imaging. Nonetheless, deep inside tissue the influence of scattering accrues to limit optical penetration. The zebrafish vasculature provides a prime example of a spatially disperse and localized structure comprising only a minute portion of the total sample volume. Figure 5a,b shows a single plane from a vascular labelled transgenic (*Tg*(*kdrl:GFP*)) (5 dpf) imaged: (a) in its entirety and (b) by pulsing the laser with varying phase delay. On the far side of the image (illuminating from the left) the signal reaching the camera is reduced owing to rejection of non-ballistic signal by the confocal slit. A ubiquitously labelled transgenic, *Tg*(*h2afva:h2afva-EGFP*), provides a more homogeneously fluorescent specimen. Figure 5c shows an image of a single plane through the head and trunk section of the zebrafish (5 dpf). The head provides the largest profile with which to test the patterning ability, however the eye attenuates the beam significantly. For further analysis, the image was limited to the bounding box shown, for which the depth into tissue is near-invariant along the *y*-axis (visible surface *x* position changing by ±10 μm). Figure 5d shows the ROI as the laser is pulsed with varying phase delay. Also shown is the *y*-averaged intensity and equivalent focal position. The fluorophore distribution remains too spatially variant to allow quantitative measures of patterning resolution, a clear movement of the intensity maxima in line with the focal position is evident until deep within tissue the ballistic content of light is negligible.

### Imaging performance

**Figure 6:**
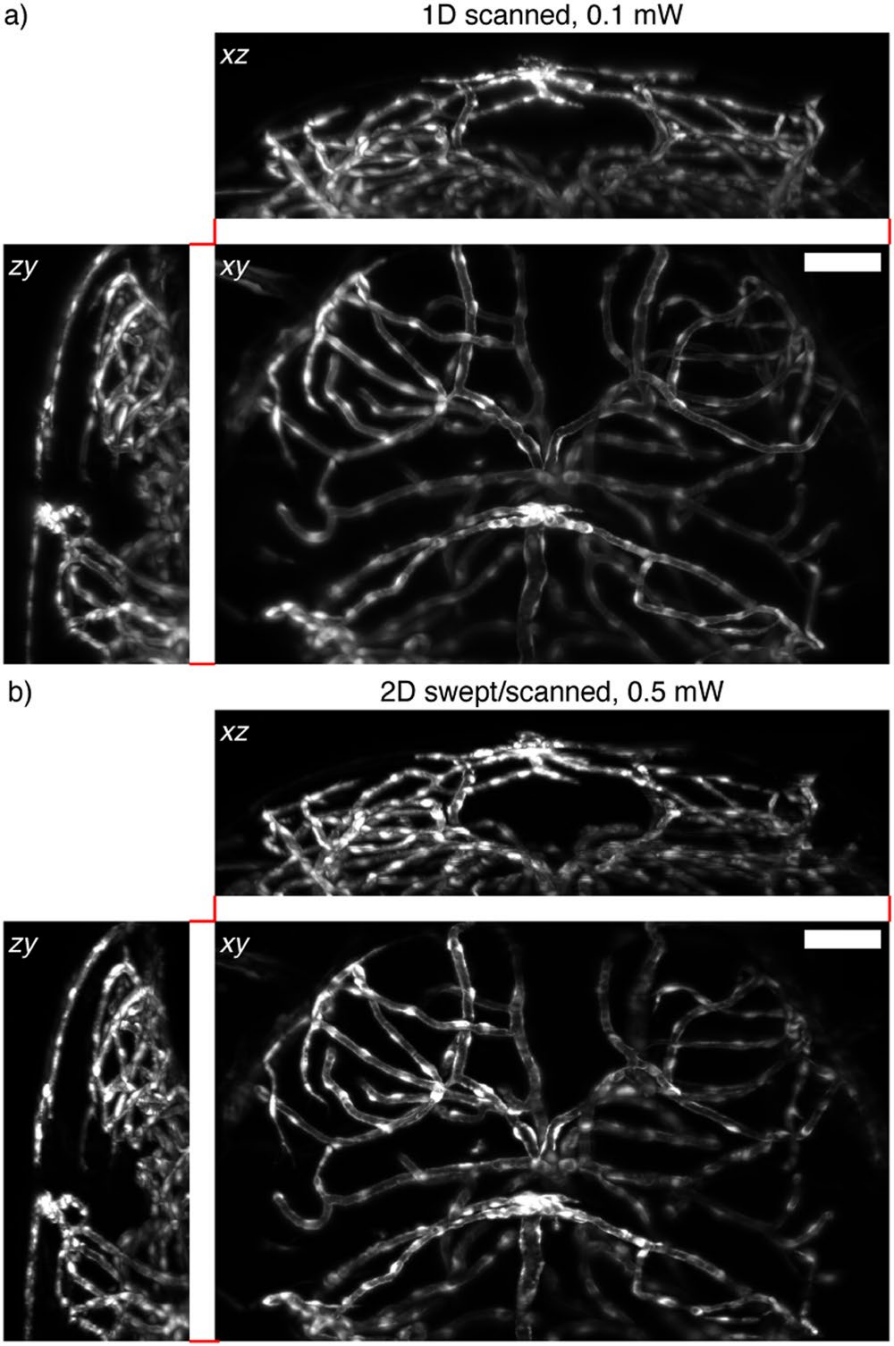
Image quality (*Tg*(*kdrl:GFP*), 5 dpf) and photobleaching (*Tg*(*h2afva:h2afva-EGFP*), 5 dpf) in zebrafish (42% TAG amplitude). All images are maximum intensity projections along the excluded axis. Scale bars = 100 μm. (**a**) 1D scanned mode, 0.1 mW laser power. (**b**) 2D swept/scanned mode, 0.5 mW laser power.

In the pursuit of improved spatial confinement of the light dose it is crucial that the image quality is not unduly affected. Figure 6a shows maximum projections from stacks of images of vascular labelled zebrafish Tg(kdrl:GFP) (5 dpf) using the 1D scanned mode at 0.1 mW laser power (measured before the illumination objective). For comparison, the same fish was imaged using the 2D swept/scanned mode with 0.1 mW and 0.5 mW laser power. For equal power the image contrast was lower than the 1D scanned mode. However, using 0.5 mW laser power delivered similar image quality on visual inspection (Fig. 6b).

We define two metrics for image quality based on (i) separability of foreground and background and (ii) spectral information content (Supplementary Note 4). Full results of the two metrics are listed in Supplementary Table 1 for each of the imaging modes in both the *x*, *y* and *y*, *z* views (to capture lateral and axial resolution respectively). This analysis confirms that a 5-fold increase in laser power for the 2D swept/scanned mode delivers similar, or even slightly superior, image quality (for metric i). However, the spectral content remains lower, owing to incomplete blocking of out of focus light by the confocal slit^31^. In all cases the *y*, *z* entropy is lower due to the intrinsically lower axial resolution but the same ordering was observed. A comparison with a static tissue phantom (hydrogel encased fluorescent beads is also provided (Supplementary Note 4), which demonstrates no loss of spatial resolution for the 2D swept/scanned mode.

The need for additional laser power is undesirable but expected. In fact, we find the scaling to be less problematic than predicted based on the NA_ill_^−1^ dependency; recall that only a 5 times increase in laser power was required for a ca. 10 times increase in NA_ill_ (approximately: NA_ill,1D_ = 0.03, NA_ill,2D_ = 0.3). This supports the logic that a wider slit is beneficial for optical efficiency. Nevertheless, this increase in laser power reflects full plane illumination, the limiting case that is abstracted from the intended purpose of plane-wise illumination patterning.

## Discussion

Herein we report a light sheet microscope capable of producing fully patterned illumination in 4D (*x*, *y*, *z*, *t*) using a 2D swept/scanned scheme and fine temporal control of the laser power. The ability to pattern along *x* is somewhat limited by spherical aberration but sufficient over typical penetration depths. Although it is not possible to extract the on-axis patterning from the images, the patterning measured from the inherently projected images at the native focus is consistent with results from simulations, suggesting that the ability to pattern the illumination approaches that expected from theory. In future, superior optics may yield a tighter focus providing superior axial resolution and patterning. However, even a moderate NA focus is difficult to maintain deep within tissue. Regardless, the confocal effect could be exploited to allow synchronous double sided illumination for greater sample coverage^32^. Adopting an aberration free remote focusing scheme to achieve patterning not marred by the focus lengthening effects of spherical aberrations may also prove a fruitful direction^33^.

The optical system reported demonstrates the principal of adaptive illumination with many of the benefits of light sheet microscopy (namely high spatiotemporal resolution and optical sectioning) and with the added benefit of being able to selectively pattern the temporally constructed light sheet. We have shown how this may be achieved by sweeping a short focus through the sample and pulsing the illumination accordingly. The power of light sheet microscopies stems from their blend of parallelization, permitting low instantaneous intensities and planar, confined illumination. The inherent tradeoff in temporally constructing a patterned virtual light sheet is the concomitant decrease in dwell time at each location in the FOV and associated increase in laser power required. However, the short line segments translate to a far greater parallelization than point scanning microscopies, which still provide the backbone of 3D biological fluorescence imaging. In this regard, the 2D swept/scanned mode was found to operate effectively at a laser power just 5 times higher than the 1D scanned mode. In the limiting case, where the entire plane is illuminated, the reported scheme is not optimal. However, in sparse samples, the total light dose could be greatly decreased using this method. A remaining challenge concerns the determination of the appropriate illumination pattern to deliver to the sample. Such a scheme will be dependent on the sample in question and abstracted from the desire to develop a generally applicable light sheet microscopy platform with this additional functionality and so we refrain from considering specific biological applications. Although some sacrifice to the confinement of illumination is necessary the degree of sectioning for the 2D swept/scanned mode approaches that of the common 1D scanned light sheet variant. With two-photon excitation, in principle, the confinement of the fluorescence excitation (and hence sectioning) and patterning resolution could be improved^23^. In any case, the microscope effectively demonstrates the concept of adaptive illumination in smart microscopy and improvements to the degree of parallelization and sectioning will come in future.

In the context of a smart microscope, the desired illumination pattern can be reconfigured and transferred to the field programmable gate array (FPGA) in real-time allowing dynamically evolving ROIs to be set between consecutive image planes to illuminate arbitrary sub-volumes. In tandem with sophisticated computer guidance such a system could be used to detect biological abnormalities and image appropriately without unnecessarily exposing other regions of the sample. In the future, an autonomous microscope could make discoveries that may otherwise go unseen, create and test hypotheses and do so with minimal invasiveness. In this regard, adaptive illumination provides just one part of the puzzle and future innovations are needed in real-time image analysis, adaptive detection hardware and intelligent data handling solutions.

## Materials and Methods

The optical and electronic configuration of the microscope is shown in Supplementary Figure 2. Unless noted all lenses are singlet plano-convex type (labeled L_x_). Briefly, the collimated output of a diode laser operating at 488 nm (Toptica iBeam Smart) is collimated and spatially filtered through a 20 μm diameter pinhole and expanded (*f*_1_ = 20 mm aspheric lens, *f*_2_ = 80 mm) to fill the useable aperture (4 mm) of a tunable acoustic gradient (TAG) lens operating at its 188 kHz resonance (TAG Optics, TAG Lens 2.5), the lens is conjugated to a galvanometric scanning mirror (Scanlab Dynaxis 3 S) by a pair of 4 *f* relay systems (*f*_3_ = 60 mm, *f*_4_ = 60 mm *f*_5_ = 75 mm, *f*_6_ = 125 mm). A final telescope consisting of a scan lens (Sill Optics, f-theta lens, *f*_7_ = 88.9 mm) and tube lens (f_8_ = 100 mm) conjugates both the scanning mirror and TAG lens with the back focal plane of a water dipping microscope objective (Olympus UMPLFLN 20 XW 20×/0.5, *f*_9_ = 9 mm), which focuses the beam into a custom-built sample chamber. The beam expansion is sufficient to deliver NA_ill_ of ca. 0.4, however system aberrations yield a focal volume better characterized as ca. NA_ill_ 0.3. For comparison, the microscope may be converted to a more conventional 1D scanned system via a pair of flip mirrors and the telescope formed by a further pair of lenses (*f*_10_ = 160 mm, *f*_11_ = 90 mm). The beam expansion was chosen to ensure that the light sheet length was approximately equal for the 1D scanned and 2D swept/scanned mode (which can be tuned by controlling the TAG lens driving amplitude).

A widefield detection path is formed by a second, orthogonally arranged water dipping objective (Nikon CFI75 16×/0.8, *f*_12_ = 12.5 mm), bandpass filter (Chroma 525/50), tube lens, (Nikon, *f*_13_ = 200 mm), and sCMOS camera (Andor, Zyla 4.2). The camera was operated at 10 fps for experiments in fluorescent dye and 40 fps for those in zebrafish. The rolling shutter of the camera was synchronized to the y-axis beam scanning to remove out of focus signal. Volumetric z-stacks were acquired by translating samples (encased and immobilized in 1.5% w/v agarose gel) through the light sheet and controlled by motors (Physik Instrumente, M-111–1DG, C-884). Samples were rotationally oriented using a rotational piezo stage (Physik Instrumente, U-651, C-867). Image stacks were oversampled in *z* (typical plane spacing 500 nm) ensuring that the optical resolution could be explored free from sampling limitations. All electronics are controlled and synchronized using an FPGA (National Instruments, NI-7841R) and all tasks are carried out in the LabVIEW and LabVIEW FPGA environments. Zebrafish larvae were anaesthetized using tricaine (150 mg/l) (Sigma-Aldrich) and mounted for imaging in agarose (1.5% w/v) extruded from a glass capillary.

### Ethical approval statement

Zebrafish larvae were handled in accordance with EU directive 2011/63/EU as well as the German Animal Welfare Act. Experiments were performed on larvae during early development only (5 days post fertilization or less). At or below this developmental stage the larvae are not defined as animals under protection under the German Animal Welfare Act. All experiments involving zebrafish larvae were carried out at the Max Planck Institute of Molecular Cell Biology and Genetics, which is licensed for zebrafish handling by the Regierungspräsidium Dresden, Reference numbers: 24-9168.24-9/2012-1 and 74-9165.40-9-2001. Zebrafish husbandry and the experimental protocols as described were approved under this remit.

### Data availability statement

All images data included in this study are available from the corresponding author upon reasonable request.

## Electronic supplementary material

Supplementary Material
