# Supplementary material for: Adaptable, illumination patterning light sheet microscopy
Source: Sci Rep. 2018 Jun 25;8:9615. doi: 10.1038/s41598-018-28036-2 (PMC6018501; doi:10.1038/s41598-018-28036-2)
Supplement: Supplementary file 1 — Supplementary Material [file 41598_2018_28036_MOESM1_ESM.docx]

**Supplementary Material for: Adaptable, illumination-patterning light sheet microscopy**

Rory. M Power ^1,2,3^, Jan Huisken ^1,2,3*^

^1^ Department of Medical Engineering, Morgridge Institute for Research, 330 N Orchard Street, Madison, Wisconsin, 53715, USA

^2^ Department of Biomedical Engineering, University of Wisconsin-Madison, 1550 Engineering Drive, Madison, Wisconsin, 53706, USA

^3^ Max Planck Institute of Molecular Cell Biology and Genetics, Pfotenhauerstrasse 108, 01307 Dresden, Germany

*Corresponding author: [jhuisken@morgridge.org](mailto:*opex@osa.org)

**Supplementary Note 1: Existing strategies for light patterning along the propagation axis (*x*)**

In order to achieve full 4D patterning of light (*x,y,z,t*) it is necessary that one is able to controllably vary the intensity along the propagation axis (*x*). With regard to the use of patterned illumination in light sheet fluorescence microscopy there are four important aspects: i) the pattern contrast that the method is able to deliver at the boundaries between the extreme cases of purely on and purely off should be maximized in order to minimize photodamage to regions containing no useful information content. ii) Illumination of a given sub-ROI should not compromise image quality at some other sub-ROI owing to the presence of diffuse out of focus signal in the resulting image. iii) The patterning process should not unduly affect the temporal resolution achievable by conventional light sheet microscopes. iv) Finally, the scheme should be as optically simple as possible to maintain much of the inherent robustness of light sheet microscopy.

Several options present themselves in this regard: these can be sub-divided as those i) based on non-linear excitation and ii) those based on interference/holography. The former case exploits the quadratic dependence of the signal on the intensity to limit signal evolved from non-focal locations. This has been illustrated in optogenetics to optically stimulate several regions of the larval zebrafish brain in parallel when combined with temporal focusing and wavefront shaping techniques ^1^. Such a scheme is interesting but optically complex. Moreover, in delicate biological samples, particularly those containing pigments, two-photon excitation is inadvisable for long term imaging, particularly given the inherently low signal rate^2^.

In the latter case, a recent theoretical study recommends exploiting interference between a pair of orthogonal but co-planar structured light sheets to deliver *x*,*y* patterning of the combined sheet ^3^. Aside from the optical complexity and added steric constraints, the reliance on interference limits this approach to very small samples or superficial tissues where the light sheets maintain their spatial structure throughout. Moreover, it is not clear that this approach allows for arbitrary patterning of the light sheet. Other partially parallel holographic methods to produce light fields patterned along the propagation direction have been reported ^4^, however, these do so with some unintended increase in intensity both above/below the plane that we wish to probe and between the longitudinal intensity maxima and so the non-ROI regions may still observe higher rates of non-linear photodamage than if each of the maxima had been illuminated sequentially.

**Supplementary Note 2: Spatial and temporal constraints on beam scanning (*y*), sweeping (*x*) and patterning (*x,y*)**

In lieu of an efficient parallelized strategy to deliver patterned illumination, while meeting the criteria given in Supplementary Note 1, we have concluded that sequential illumination of sub-ROIs is preferable. In section 2 B. it was asserted that a combination of scanning the beam in *y* with a galvanometric (galvo) scanning mirror and sweeping the beam in *x* using a TAG lens is sufficient to produce the light sheet within the exposure time of the camera but here we quantitatively illustrate this claim (see Fig.1 for a description of the microscope coordinate geometry).

Delivering 2D plane-wise spatial patterning requires not only that the virtual light sheet be produced within the exposure time of the camera (typically 10 – 100 ms) but additionally requires a high degree of temporal control of the illumination intensity. The galvo mirror itself is capable of reproducing a driving signal containing frequencies of several hundred Hz over the small angles. (± ca. 1.5°) required for covering a field of view height (*y*, ca. 800 μm) and so is able to faithfully reproduce the triangle wave driving signal at the ca. 40 frames per second imaging speed used throughout. This has been confirmed by ensuring that the beam and active area of the camera remain co-aligned throughout the acquisition process.

The TAG lens operates in a resonant mode with the frequency and amplitude determining the optical power and useable aperture. These characteristics will inevitably place some constraint on the necessary modulation bandwidth and although a lower resonant frequency for the TAG lens operation relaxes these constraints, the choice of resonance frequency is more dependent on optical concerns. The focal displacement, d*x*_TAG_, elicited by the TAG lens refocusing is given by:

$dx_{TAG}=-\frac{n_{imm}.f_{obj}^{2}}{f_{TAG}.M^{2}}$ (S1)

Where n_imm_ is the immersion media refractive index, f_obj_ and f_TAG_ are the focal lengths of the illumination objective lens and TAG lens, respectively, and M is the magnification from the TAG lens to the illumination objective back focal plane. The full scan range will correspond to the difference in *x* for the shortest positive and negative focal lengths of the lens (i.e. the highest absolute optical power cases). From equation S1. it becomes clear that to maximize the refocusing range of the microscope, one should take care to minimize the magnification between the TAG lens and objective back aperture. This runs counter to the need to expand the beam to obtain a moderate illumination NA sufficient to deliver a high degree of spatial patterning. For this reason, the useable aperture of the TAG lens comes to disproportionately govern the effective optical power of the refocusing system. In this regard, the aperture corrected optical power, OP_aperture,_ is given by the product of the optical power and area of the aperture. For a small aperture one requires high magnification through the various 4*f* systems to appropriately fill the illumination objective back aperture. This decreases the scan range quadratically and so a lower absolute optical power over a larger aperture is usually preferable.

Using the aperture corrected value, the largest scan range is given by the resonance at 188 ± 1 kHz, (up to 20 diopters at 4 mm aperture or OP_aperture_ = 10.1 × 10^-3^ m). This defines a period of 5.3 μs to scan the focus back and forth through the effectively extended DOF. The total refocusing range observed (490 ± 0.8 μm at 42% TAG lens amplitude, 737 ± 0.8 μm at 61 % amplitude) was fairly consistent with that predicted from equation 1 once the TAG lens had established a stable resonance (633 μm at 42% TAG lens amplitude, 873 μm at 61 % amplitude). This calculation assumes a linear variation in optical power with amplitude and uses the manufacturer quoted optical power at various driving amplitudes (10 or 20 diopters at 32, 72% amplitude respectively). The discrepancy owes to the linear interpolation and the fact that the stabilized resonance exhibits slightly lower optical power than immediately after tuning to the resonance frequency.

Assuming a total exposure time of 25 ms (used throughout all experiments), the TAG lens relayed to the object will focus from one limit of its optical power to the other (i.e. across the field of view once) ca. 9400 times per image. Correspondingly, complete coverage of the field of view during the y-scanning is trivial and each of the 2048 pixel lines of the camera will be swept ca. 4.5 times.

Of course, without appropriate temporal control of the laser pulses, the patterning resolution will be dominated by the modulation bandwidth. Using a diode laser and field programmable gate arrays (FPGA), allowed digital modulation at up to 40 MHz and analog modulation at up to 1 MHz. In the digital case, for the more typical example 490 μm scan range at 42 % amplitude, the modulation bandwidth is sufficient to provide ca. 4.7 μm resolution on the spatiotemporal patterning ability along *x*. This is comparable with the Rayleigh range of the illumination beam at the native focus, however, even when the two factors are combined the patterning resolution is sufficient to cover tissues spanning tens to hundreds of microns. The analog modulation bandwidth is far lower, and sufficient only to allow x patterning with a spatial resolution of ca. 186 μm. For this reason, analog modulation is currently limited to patterning in *y*.

**Supplementary Note 3: Simulations of 1D scanned and 2D swept/scanned light sheets**

The first set of simulations (Fig. 4 a), b) was performed as follows: The spatial intensity profile of a Gaussian beam (NA = 0.3, n_imm_=1.33, λ_0_ = 488 nm) is simulated over a volume of 200 × 15 × 15 μm^3^ (0.25 μm step size) utilizing a simple Gaussian optics treatment. The resulting light sheet produced by scanning this beam along *y* was simulated in the absence of a confocal slit (widefield mode) by convolution with a rectangular function (equal to 1 if *x* or *z* = 0 and equal to 0 otherwise.) To simulate aperturing of the beam by a confocal slit with width, w_confocal,_ the Gaussian beam intensity was set to zero where -½.w_confocal_ < *y* < ½.w_confocal_ (w_confocal_ = 7.25 μm in object space for Fig. 4 a, b). The apertured beam was then convolved with the same rectangular function.

To simulate the patterning resolution apparent from the images we also performed z-projections of the various light sheets. To simulate the 3D image formation in homogenously fluorescent media with the detection plane at *z* = 0, one should consider that the system PSF (from both illumination and detection components) at each point is equal to the product of the plane-wise illumination and detection PSFs. Mathematically this is equivalent to the product of the simulated light sheet intensity (I(*x,y,z*)) and the detection PSF convolved with a plane-wise rectangular function (equal to 1 if *z* = 0 and equal to 0 otherwise, i.e. to simulate the sum of detection PSFs at each point within the detection plane.) Since the latter term is itself uniform throughout the entire volume (since the integrated intensity of the detection PSF is equal over any plane orthogonal to the propagation axis) one may dispense with considerations relating to the detection PSF and the image formation is mathematically equivalent to the *z*-projection of the illumination PSF along z. As such, the z-projected nature of the images can be explored simply by a summed-intensity projection along z.

A second set of simulations (Fig. 4 c), d) were carried out, differing only in that the beam was convolved with a rectangular function (equal to 1 if *z* = 0 and equal to 0 otherwise) to reflect the scanned (*y*) and swept (*x*) nature of the light sheet (as opposed to simply scanning as in the first set of simulations). In this case, it is more intuitive that the summed projection along *y* can be used to explore the rejection of light, due to the presence of the confocal slit, across the height of the light sheet.

**Supplementary Note 4: Image contrast and information content in optically inhomogeneous media**

Switching between the 1D scanned and 2D swept/scanned imaging modes requires some realignment and recalibration. This is due to the need to change NA_ill_ by a factor of ca. 10 between the 1D scanned and 2D swept-scanned imaging modes and so requires individual beam expansion and optical pathways. During the time taken to complete realignment/recalibration the zebrafish undergoes small but noticeable developmental changes. Even following image registration, using a line profile to illustrate image contrast is fraught with challenges as one cannot say that the position or brightness of the vasculature remains the same. Nevertheless, the overall fluorophore content should be fairly constant and instead to better quantify image quality, two metrics were used: i) the separability of signal and background was determined using a local Otsu’s threshold to separate the foreground and background and subsequently taking the difference between the mean intensities of the two populations. One would expect that an image contaminated by out of focus signal would feature a higher background and a lower separation accordingly. Taking the 1D swept/scanned case as the exemplar, all other values are normalized relative thereto. ii) The spectral entropy provides a metric of image information content. Images with finer details contain higher frequencies in their spectrum and therefore a higher spectral entropy. Since image entropy is partially logarithmic the values reported are absolute (Supplementary Table 1).

**Supplementary Table 1: Image quality metrics associated with the images of a zebrafish larva shown in Fig. 6**

| Image Orientation: | *xy* | |  | *yz* | |
| --- | --- | --- | --- | --- | --- |
| Image Mode | Foreground-Background Separation | Spectral entropy |  | Foreground-Background Separation | Spectral entropy |
| 1D scanned (0.1 mW) | 1.000 | 7.915 |  | 1.000 | 5.346 |
| 2D swept/scanned (0.1 mW) | 0.178 | 6.465 |  | 0.163 | 4.369 |
| 2D swept/scanned (0.5 mW) | 1.082 | 6.941 |  | 1.096 | 4.467 |

Supplementary Table 1: Image quality metrics for 1D scanned and 2D swept/scanned imaging modes in zebrafish larvae.

Due to scattering in tissue and its interaction with light rejection by the confocal slit, it is crucial that the various imaging modes are tested in the relevant biological context. However, one may similarly explore the image quality in a truly static tissue phantom provided by fluorescently labelled beads dispersed in hydrogel. By using beads smaller than the diffraction limit of the detection system this additionally facilitates comparison of the point spread functions (PSFs). The results of these studies can be found in Supplementary Tables 2 and 3. We found similar patterns as in tissue. Both foreground-background separation and spectral entropy increase with higher laser power. Notably the small loss of spectral entropy observed in tissue for the 2D swept/scanned mode was not apparent, although the separability did decrease slightly. We suggest that separability may have been higher in tissue relative to the 1D swept/scanned mode owing to the rejection of scattered light (which would otherwise contribute to background signal). In the far more weakly scattering hydrogel encased bead samples this additional background suppression (relative to the 1D scanned mode) does not occur. Nevertheless, in both cases we find that the foreground and background are highly separable and that the frequency content is sufficient to achieve the required resolution.

**Supplementary Table 2: Image quality metrics from hydrogel encased fluorescent beads**

| Image Orientation: | *xy* | |  | *yz* | |
| --- | --- | --- | --- | --- | --- |
| Image Mode | Foreground-Background Separation | Spectral entropy |  | Foreground-Background Separation | Spectral entropy |
| 1D scanned (0.1 mW) | 1.000 | 10.63 |  | 1.000 | 8.338 |
| 2D swept/scanned (0.1 mW) | 0.569 | 5.13 |  | 0.327 | 6.729 |
| 2D swept/scanned (0.5 mW) | 0.800 | 10.36 |  | 0.744 | 8.363 |

Supplementary Table 2: Image quality metrics for 1D scanned and 2D swept/scanned imaging modes in hydrogel encased fluorescent beads.

Analysis of the PSF dimensions allows the resolving power of the various modes to be explored further. PSF analysis was performed using the same bead samples and PSFJ ^8^. In all cases an averaged PSF was calculated by fitting to > 60 individual bead sub-volumes and taking a lateral resolution as the mean of the two lateral dimensions (*x,y*). All *x*-positions were included although spherical aberration of the light sheet may result in some axial elongation of the PSF along *x*. This was not immediately apparent from the results of Supplementary Table 3 but may account for the larger variability for the 2D swept/scanned mode. Previous studies have sought to illustrate that the 2D swept/scanned approach leads to superior axial resolution, and under one-photon excitation this has required the use of a very narrow confocal slit (limiting optical efficiency) and a more tightly focused beam (limiting field of view, owing to the scaling laws of equation S1, and general robustness) ^9^. Improving the axial resolution is beyond the scope of this paper, however it is crucial that the gains to patterning ability do not come at the cost of sacrifices to either axial or lateral resolution. From Supplementary Table 3, it is clear that the resolution of the 1D scanned and 2D swept/scanned modes are equal within the variability between the various PSF fittings.

**Supplementary Table 3: PSF dimensions from hydrogel encased fluorescent beads**

| Image Orientation: | Lateral (*xy*) /nm | |  | Axial (*z*) /nm | |
| --- | --- | --- | --- | --- | --- |
| Image Mode | Resolution | Variability |  | Resolution | Variability |
| 1D scanned (0.1 mW) | 606 | 6.5 |  | 2740 | 190 |
| 2D swept/scanned (0.1 mW) | 629 | 29 |  | 2650 | 227 |
| 2D swept/scanned (0.5 mW) | 631 | 23 |  | 2790 | 222 |

Supplementary Table 3: Image quality metrics for 1D scanned and 2D swept/scanned imaging modes in hydrogel encased fluorescent beads.

**Supplementary Figure 1: Pattern contrast in optically homogeneous media**


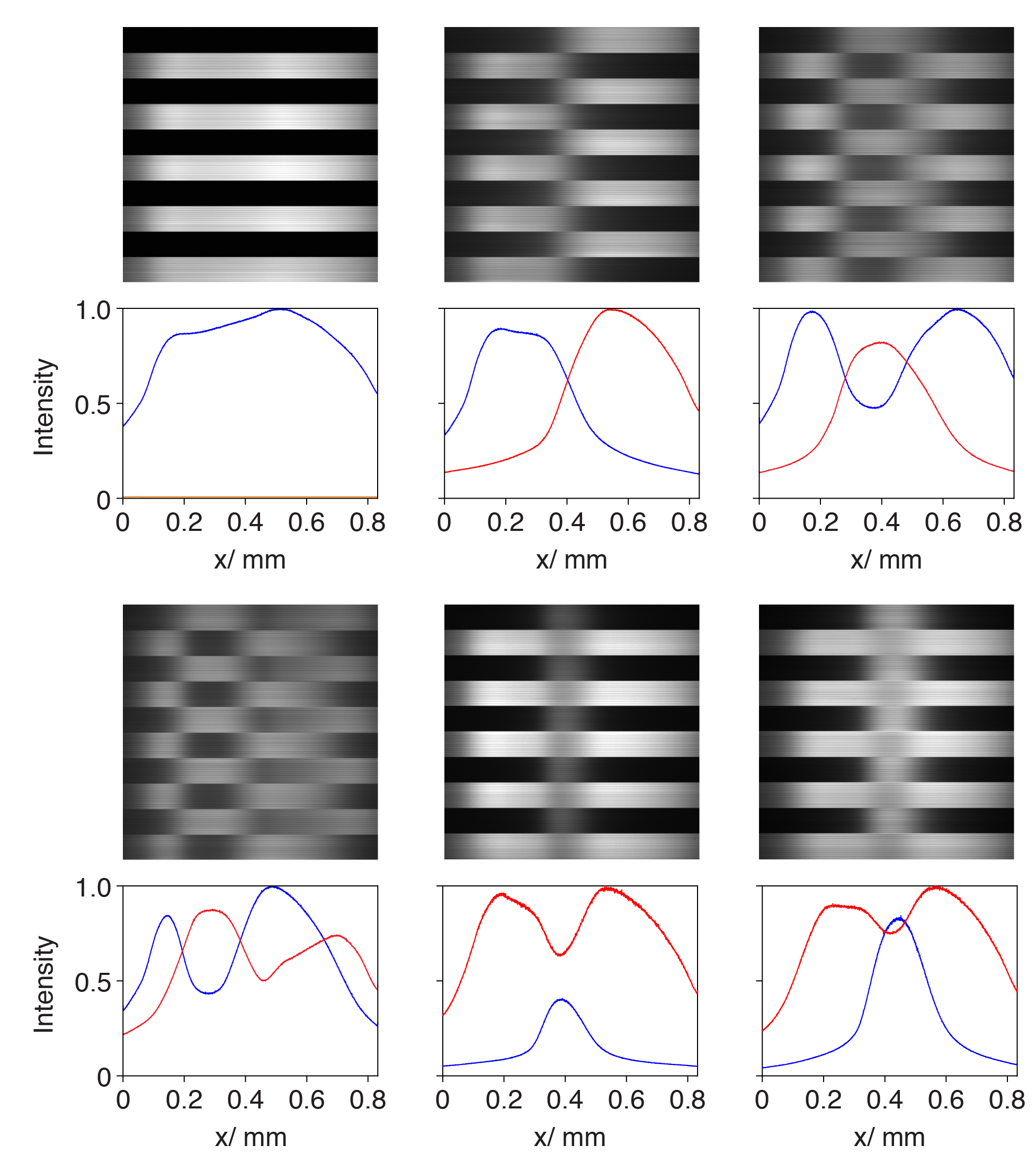


Supplementary Figure 1. Line profiles for the various patterned images from Figure 4 b), c). In each case the red line corresponds to the average line pattern of the top 200 pixel rows (*y,* vertical, ROI height/location denoted by the red bar next to the image) which have been illuminated using the same line pattern along *x*. The blue line corresponds to the average of the bottom 200 pixel rows (ROI height/location denoted by the blue bar next to the image).

**Supplementary Figure 2: Optical and electronic experimental configuration**


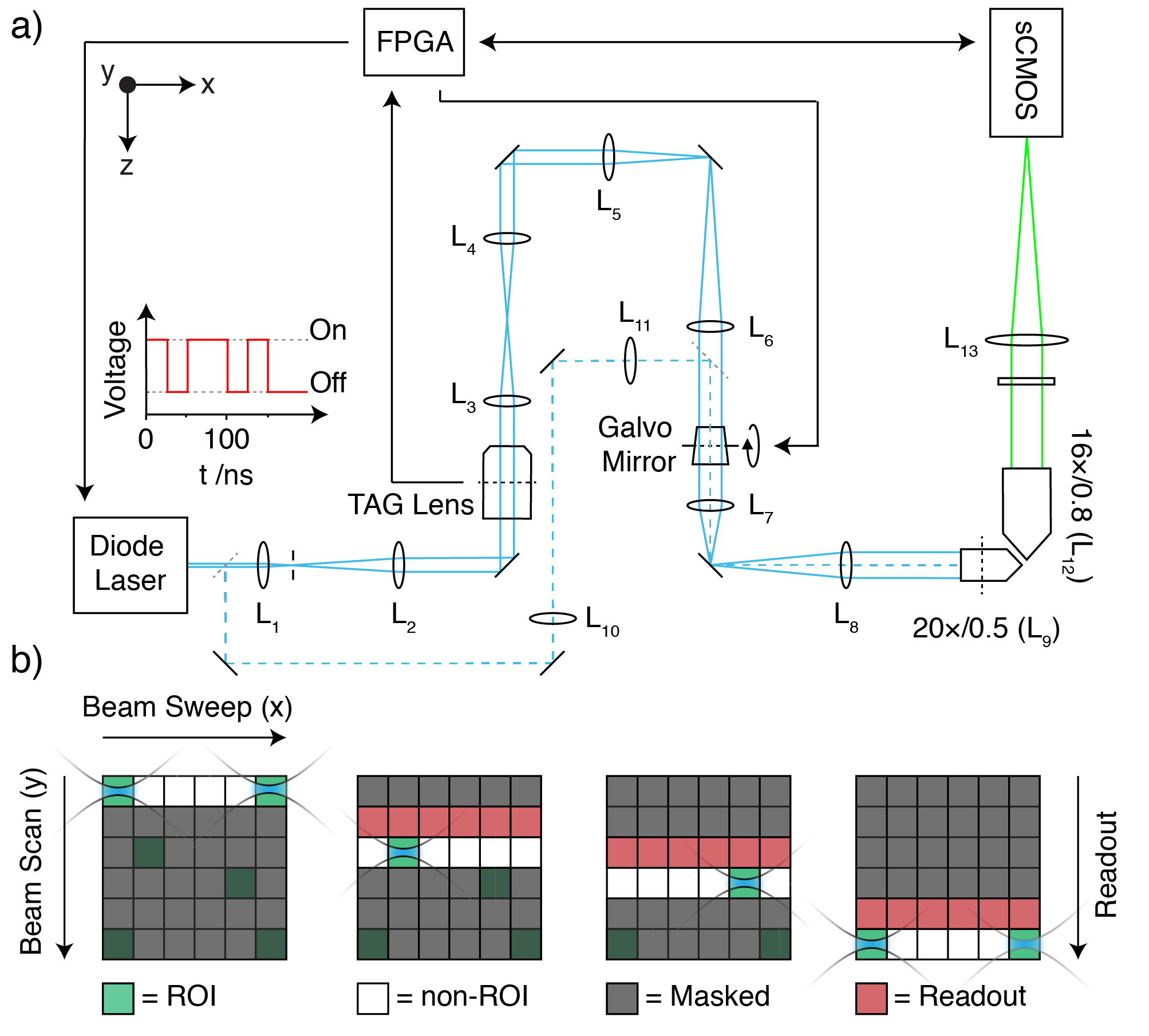


Supplementary Figure 2. Experimental configuration. a) The optical pathways of the microscope and electronic control of the various hardware elements. The TAG lens and galvanometric scanning mirror are conjugated to the back focal plane of the illumination objective via 4*f* systems to produce 2D swept/scanned light sheets. Flip mirrors allow the TAG lens to be bypassed in the 1D scanned mode. Custom software running on an FPGA provides synchronization the electronic components. The FPGA takes a pulsed input from the TAG lens, demarcating the start of an oscillation period and providing the master timing input (i.e. governing the trigger timing of all other components). Additional inputs from the camera are required to determine whether the camera is ready to be triggered for each frame, which row of the camera is being read out (for synchronization with the scanning mirror) and whether the camera exposure has completed. The FPGA provides outputs to the scanning mirror (analog), laser pulsing (analog, digital) and camera trigger (digital). Since the onboard memory of the FPGA is limited to 8 MB, the illumination pattern has to be loaded line by line from the host PC to the FPGA. This process is sufficiently fast to allow each of the 2048 pixel lines (*y* dimension*)* of the camera to be illuminated with a unique pattern and for each image acquisition to similarly have a unique pattern. b) Confocal line detection allows diffuse out of focus background arising from the swept beam tails to be removed by synchronizing the y beam-scanning to a line-like exposed region on the camera. Sub-ROIs within the same x-line are illuminated sequentially but with sufficient rapidity that the exposure region may be considered static (the time taken for the readout row to move across one slit width comprises ca. 50-100 TAG lens periods).

**Supplementary References**:

1. Hernandez, O. *et al.* Three-dimensional spatiotemporal focusing of holographic patterns. *Nat. Commun.* **7,** 11928 (2016).

2. Power, R. M. & Huisken, J. A guide to light-sheet fluorescence microscopy for multiscale imaging. *Nat. Methods* **14,** 360–373 (2017).

3. Manton, J. D. & Rees, E. J. triSPIM : light sheet microscopy with isotropic super-resolution. *Opt. Lett.* **41,** 4170–4173 (2016).

4. Antonacci, G., Domenico, G. Di, Silvestri, S., DelRe, E. & Ruocco, G. Diffraction-free light droplets for axially-resolved volume imaging. *Sci. Rep.* **7,** 17 (2017).

5. Chmielewski, A. K. *et al.* Fast imaging of live organisms with sculpted light sheets. *Sci. Rep.* **5,** 9385 (2015).

6. Fahrbach, F. O., Voigt, F. F., Schmid, B., Helmchen, F. & Huisken, J. Rapid 3D light-sheet microscopy with a tunable lens. *Opt. Express* **21,** 21010–21026 (2013).

7. Fu, Q., Martin, D. L., Matus, D. Q. & Gao, L. Imaging multicellular specimens with real-time optimized tiling light-sheet selective plane illumination microscopy. *Nat. Commun.* **7,** 11088 (2016).

8. Theer, P., Mongis, C. & Knop, M. PSFj: know your fluorescence microscope. *Nat. Methods* **11,** 981–982 (2014).

9. Dean, K. M. & Fiolka, R. Uniform and scalable light-sheets generated by extended focusing. *Opt. Express* **22,** 26141–26152 (2014).
